# Evaluation of China’s live streaming e-commerce industry policies based on a three-dimensional analysis framework

**DOI:** 10.1371/journal.pone.0301451

**Published:** 2024-05-14

**Authors:** Bing Wang, Chenhao Tong, Tinggui Chen, Jianjun Yang, Guodong Cong

**Affiliations:** 1 School of Artificial Intelligence and Electronic Commerce, Zhejiang Gongshang University Hangzhou College of Commerce, Hangzhou, China; 2 School of Statistics and Mathematics, Zhejiang Gongshang University, Hangzhou, China; 3 Department of Computer Science and Information Systems, University of North Georgia, Oakwood, Georgia, United States of America; 4 School of Business Administration, Zhejiang Gongshang University, Hangzhou, China; University of Naples Federico II: Universita degli Studi di Napoli Federico II, ITALY

## Abstract

As an emerging business modality and Internet format, live streaming e-commerce has developed rapidly since its emergence in 2016, especially since the outbreak of the COVID-19 epidemic in late 2019, when an increasing number of businesses from other industries attracted participation. However, with the development of the live streaming e-commerce industry, the industry’s market environment is becoming increasingly chaotic. Therefore, during this period, government departments continuously formulate and implement relevant industry policies. In order to exploring the cooperation network structure, policy content distribution, and implementation effectiveness characteristics among publishers, this paper constructs a three-dimensional analysis framework of policy from the perspective of policy tools, policy effectiveness evaluation and policy publishers. The results show that in terms of policy tools, the overall structure of policy tools in the live streaming e-commerce industry is unreasonable, and different types of policy tools are significantly diverse. The proportion of environmental policy tools is greater than that of demand-based and supply-based policy tools, accounting for 62.97%, and among them, the tools related to industry regulation and management account for the largest proportion of the total, which greatly suppresses the enthusiasm of various entities in the industry for development. In terms of policy effectiveness evaluation, most of the policies do not formulate detailed long-, medium-, or short-term goals, nor are the policy priorities, incentive measures, or action modes perfect, indicating that the government’s pushing and pulling forces for the live streaming e-commerce industry are insufficient. Finally, in the subject dimension of policy release, the synergy of relevant subjects is constantly improving, but there is also a phenomenon of over-concentration in the synergistic departments.

## 1. Introduction

Currently, with the development of the Internet and the popularization of mobile devices, streaming media commerce such as Taobao live streaming, Sina Weibo live streaming, and Tiktok live streaming, has developed rapidly. In 2016, the live streaming e-commerce industry officially developed from mature e-commerce, social commerce platforms, and modern logistics systems. Subsequently, against the background of seeking new economic growth, with its outstanding features, such as a low entry threshold, a wide range of participation, and a large scale of influence, the live streaming e-commerce industry has further developed. According to *the Statistical Report on the Development of China’s Internet Network* released by the China Internet Network Information Center, taking the "Double 11" period as an example, during the "Tmall Double 11" period, the turnover of 62 Taobao broadcast rooms exceeded 100 million yuan, 632 Taobao broadcast rooms reached more than 10 million yuan, and the turnover of new anchors increased by 345% year on year; the number of merchants of Douyin participating in the "Double 11" activity increased by 86% year on year, and 7667 broadcast rooms exceeded one million yuan; the number of buyers participating in Kuaishou activities has increased by more than 40 percent year on year. This emerging consumption model has great market potential and has become one of the breakthroughs needed to achieve new economic growth.

Despite in the process of the development of the Live Streaming E-Commerce industry, many chaos behaviors has been disturbing the market environment, it is undeniable that live streaming e-commerce clearly plays an active role in boosting the economy, alleviating poverty, and resuming work and production. Therefore, to become a mature and standard business model similar to like traditional e-commerce, live streaming e-commerce has to go through a long process. In addition, accelerating its development requires the joint efforts of consumers, countries, practitioners, and other parties. From January 2016 to mid-2022, the implementation of industry-related policies came with the development of the live streaming e-commerce industry. An in-depth analysis of the suitability and scientific basis of these related policies will promote the healthy development of the industry. Furthermore, through quantitative analysis of policy texts, the analysis results of policies can be more precise, and it is easier to make targeted recommendations.

However, there are studies on the policies of the live streaming e-commerce industry. For example, Guo et al. [1] proposed that live streaming e-commerce had become an important driver of global trade, but limited attention had been given to this area. Sun et al. [2] also noted that although people were increasingly interested in live streaming e-commerce, relevant research was still limited, and scholars mainly analyzed the development of the live streaming e-commerce industry based on the behavioral intentions of consumers. The implementation of relevant industry policies significantly promotes the development of the live streaming e-commerce industry. Based on this, to further broaden the research ideas and fully learn from the quantitative analysis research framework of policy texts in other fields, this paper conducts a quantitative analysis of the relevant policies during the development of the live streaming e-commerce industry. From the perspectives of policy tools, policy effectiveness evaluation, and policy publishers, this paper constructs a three-dimensional analysis framework of policies through using policy tool coding, a policy modeling consistency (PMC) index evaluation system, and a social network model to quantify the relevant policy texts of the live streaming e-commerce industry, and revealing the nature, characteristics, and content of these policies and provide effective suggestions for the formulation of future policies in the industry.

The remainder of this paper is organized as follows: Section 2 is a literature review. Section 3 describes the research idea and framework of this paper. Section 4 describes the collection of policy texts and the statistical methods used. Section 5 presents the analysis results of the constructed three-dimensional analysis framework. Section 6 summarizes the full text and provides policy recommendations.

## 2. Literature review

Ever since the initial rise of live streaming e-commerce in 2016, it has been particularly eye-catching in accelerating the resumption of work and production, poverty reduction, and employment, creating marketing miracles under the promotion of many people. In the context of the current new business format of the Internet economy, to determine new economic growth points, the government should pay attention to the problems and risks existing in the online live streaming e-commerce industry. Therefore, since 2016, the government has issued a series of industry-related policies striving to explore a standardized and normalized development path for the live streaming e-commerce industry. This section discusses the related literature in two main aspects: the factors affecting the development of the live streaming e-commerce industry and the quantitative analysis of the policy texts.

(1) Factors that impact the development of the live streaming e-commerce industry

With the upgrading of Internet technology and the popularization of mobile devices, live streaming e-commerce has developed rapidly. Several typical streams of literature are as follows: Xu et al. [3] proposed that the development of traditional e-commerce was evolving. As an upgrade of traditional e-commerce, live streaming e-commerce has great potential for using various types of emerging novel applications to enhance customer participation and achieve greater economic value. Busalim and Hussin [4] proposed that this innovative live-streaming marketing approach would transform traditional e-commerce from a product oriented shopping environment to a social, hedonic and customer-centered environment, enriching traditional business in various ways. Therefore, as a booming emerging field, many scholars have studied the live streaming e-commerce industry. Yu et al. [5] explored the relevant factors that affected the purchase intentions or participation in live streaming e-commerce. This study empirically proved that the motive for socialization strongerly correlated with gift-giving behavior, which was considered the commoditization of a viewer’s social interaction while consuming media. Through a questionnaire survey of live viewers, Xu et al. [6] used the purchase intention model constructed by empirical testing, identified how social capital affected purchase intention in live streaming e-commerce, found that the streamer’s professionalism, the reciprocal expectation of live streaming, and the viewer’s parasocial relationship could effectively increase the viewer’s purchase intention. Zhang et al. [7] used stimulus-organism-response (SOR) model, constructing a research framework for the influence of knowledge sharing on consumer-brand relationship in virtual communities, finding that knowledge-sharing quality has significant positive effects on the sense of virtual community, as does the sense of virtual community on the consumer-brand relationships. Cai et al. [8] examined live streaming e-commerce, conceptualizing it as a type of online shopping that incorporated real-time social interaction, and examined the relationships between hedonic and utilitarian motivations and shopping intention; the authors found that hedonic motivation was positively related to celebrity-based intention and that utilitarian motivation was positively related to product-based intention. However, Apiradee and Nuttapol [9] suggested that research pertaining to the live streaming context was still at a nascent stage, and most related research has been conducted in the form of a survey to describe the characteristics of live streaming and consumer motivations to participate in it. Qing and Jin [10] also showed that although people were increasingly interested in live streaming e-commerce, related research was still limited, and most related research on live streaming e-commerce was based on consumer decision-making models. Few scholars currently focus on the analysis of policies in the live streaming e-commerce industry. In fact, in the development of the live streaming e-commerce industry, national government departments are constantly issuing relevant policies to promote the healthy and normal development of the industry. Therefore, quantitative research on these policy texts can be used to analyze the current policies related to the live streaming e-commerce industry effectively and put forward corresponding optimized suggestions to provide a scientific basis for the following policy formulation.

(2) Quantitative analysis of policy texts

Quantitative analysis of policy texts is the most pivotal link in formulating and managing public policies, and it is also the basis for rationally allocating policy resources. Generally, by using different quantitative models and technical means to conduct a comprehensive analysis of specific policies, one can not only make scientific judgments on the policies but also test the actual effects of policy formulation and implementation. At present, scholarly research on quantitative policy texts has focused on policy content analysis, policy system improvement, and policy effect evaluation. For example, Zeng and Yang [11] used Nvivo12 qualitative software to conduct grounded research and qualitative text analysis on 20 Chinese cultural and creative industry development policy texts, and summarized three core elements that promoted the development of China’s cultural and creative industry: “development task”, “development goal” and “development guarantee”. Guo and Niu [12] started by classifying policies and the synergistic effect of policy types and policy tools and constructing a collaborative analysis model of policy function to study collaborative research between science and technology policy types and policy tools. Finally, it was suggested that the formulation of science and technology policy should closely address the background of the times and the characteristics of the policy types, and the corresponding policy tools should be synthetically selected to match its applicability. Cheng et al. [13] used combined interviews and investigator field observations to analyze and evaluate the implementation effect of the “Link Policy” from the perspectives of participants and investigators. The results showed that the participants recognized and supported improving rural living conditions and coordinating urban-rural development, but the Link Policy failed to achieve the objectives of preserving farmland, protecting farmers’ land use rights and interests, and facilitating agricultural production. At the same time, several scholars have analyzed the government policies through the two-dimensional analysis framework of “policy goals—policy tools”, the three-dimensional analysis framework of “policy subjects—policy objects—policy goals”, and the four-dimensional research framework of “policy subjects—policy objects—policy objectives—policy tools” and provided relevant suggestions. Based on content analysis, Wang et al. [14] evaluated and analyzed the effect of the new energy vehicle policy from two dimensions: the policy instrument types and the semantic structure characteristics of the policy texts. The results showed that the most frequently used policy instrument was regulatory, and the main policy objective was the demand-pull. Zhang et al. [15] constructed an analytical framework from the dimensions of policy instruments, policy targets, and policy strength to conduct a quantitative analysis of 30 talent policy texts issued by the Sichuan Provincial Government. The results showed that policy targets focused on talent innovation and talent efficiency but paid less attention to talent flow, and policy strength was in line with strategic adjustments; however, these policies were not sufficiently sustainable. Wang [16] explored the sustainable evolution law of China’s cloud manufacturing policies from the perspectives of policy issuing departments, policy focus topics and policy tools. The results showed that the evolution of cloud manufacturing policies exhibited obvious stage characteristics; it involved three main stages, namely, “encouraging development-top level design-implementation guidance”; China’s cloud manufacturing industry was still in the development stage; and it was urgent to introduce policies that directly affect the industry.

In general, most of the relevant research is based on the quantitative analysis of policy texts from the perspective of policy tools, but little consideration has been given to evaluating policy effectiveness. Although some scholars have expanded the analysis perspective and added other dimensions, the main body still revolves around policy tools. At the same time, the research on relevant policies in the live streaming e-commerce industry is still in its infancy, and there is no systematic quantitative analysis of the text. Based on this, to broaden the research ideas of quantitative analysis of relevant policies in the live streaming e-commerce industry and analyze the rationality of current industry policies, this paper constructs a three-dimensional analysis framework of relevant policies in the live streaming e-commerce industry from three dimensions: policy tools, policy effectiveness evaluation, and policy publishers. First, according to the content of the live streaming e-commerce policy, policy tools suitable for this industry are classified. Second, by integrating the multi-dimensional perspective of policy text quantification in other fields and combined with the text mining results of live streaming e-commerce industry policy, the variables at all levels in the PMC index model are determined; thus, the pros, cons, and internal consistency of a certain policy can be analyzed from various dimensions. Obtaining the original index data for policy evaluation through text mining largely avoids subjective evaluation and improves the precision of the analysis results. Finally, social network analysis is adopted to quantitatively analyze the characteristics of the policy release subject and its cooperation network, which is conducive to clarifying the centrality of the policy publishers and the degree of coordination among the subjects and improving the policy system of the live streaming e-commerce industry.

## 3. Research design

**Fig 1:**
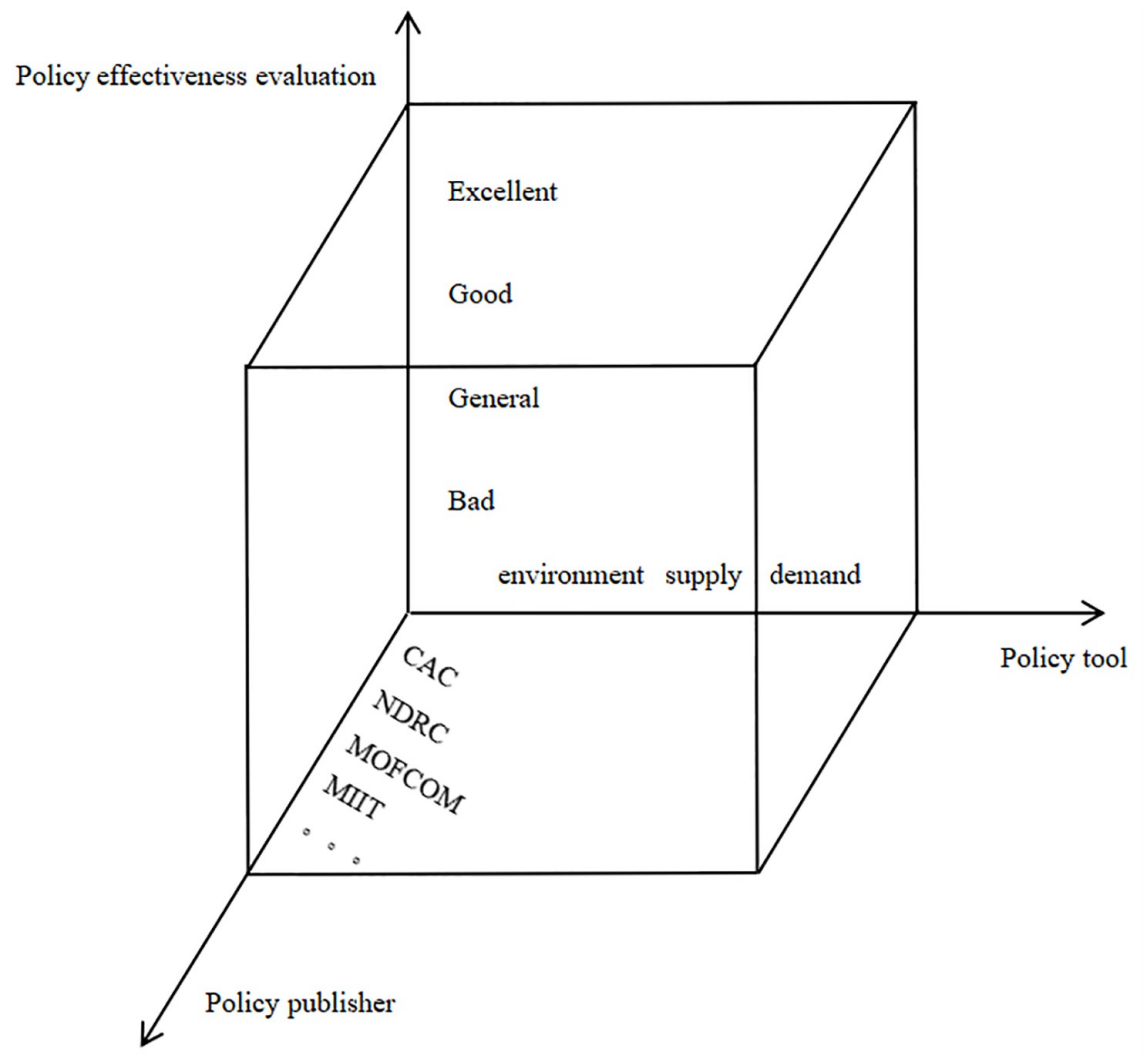
Three-dimensional analysis of relevant policies in the live streaming e-commerce industry.

Although the existing policy evaluation studies inspire this paper, most of these studies examine the use of tools from the perspective of policy tools without considering the policy path, meaning that they ignore the methods used to solve specific problems. For example, in some studies from the perspective of policy tools, the statistical analysis of supply-based tools, demand-based tools, and environment-based tools can reflect the basic situation of policies to a certain extent, but the statistical analysis of policy tools fails to fully consider the particularity of specific policies and cannot truly reflect the objective situation. Therefore, this paper conducts a comprehensive analysis of live streaming e-commerce policies and objectively evaluates them. The idea is shown in Fig 1.

**Fig 2:**
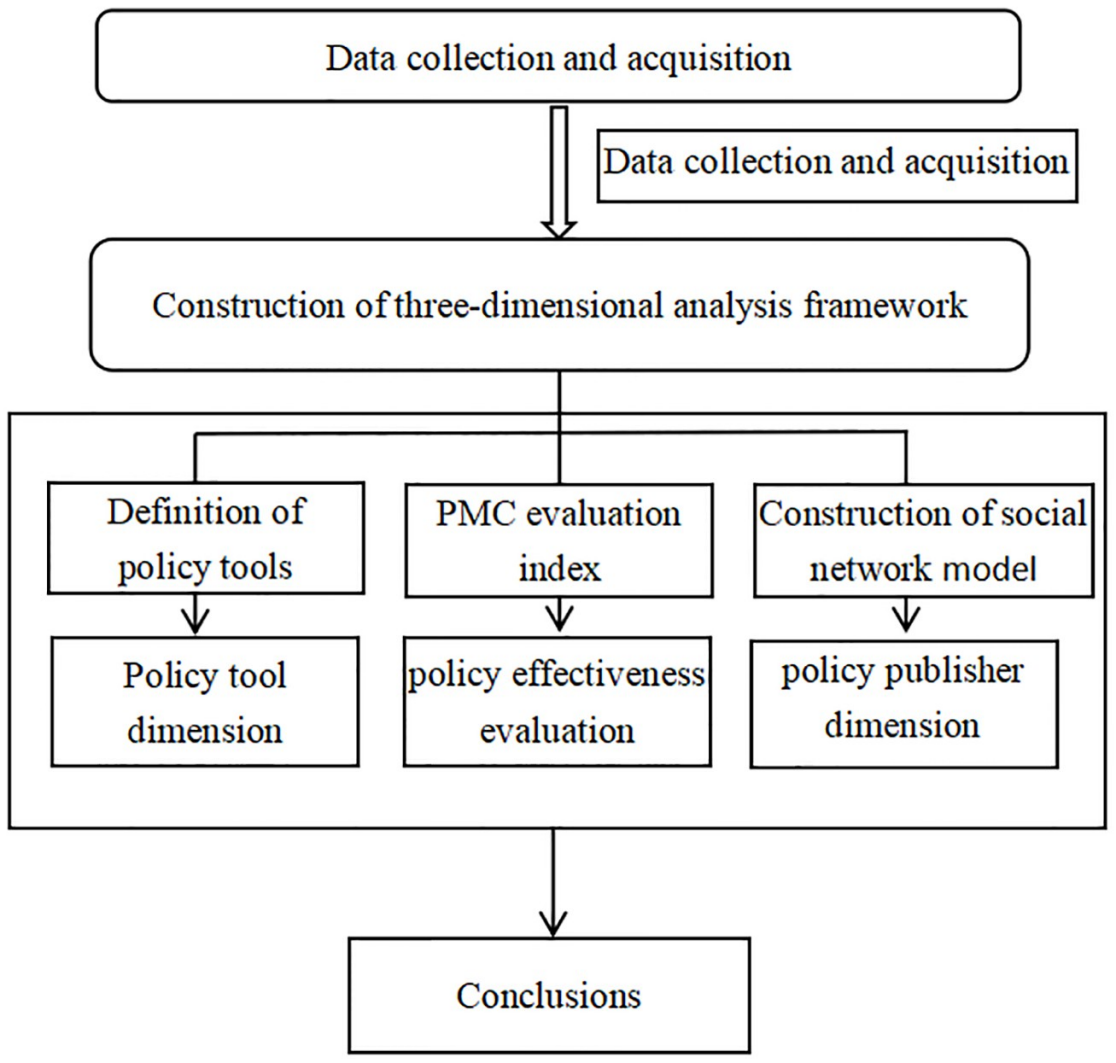
Research framework.

As shown in Fig 1, this paper constructs a three-dimensional analysis of relevant policies in the live streaming e-commerce industry from three mutually supportive and complementary dimensions: policy tools, policy effectiveness evaluation and policy publishers. In terms of policy tools, this paper divides policies into three categories: supply-based, demand-based, and environment-based. Then, according to the content of the policy text, the policy tool categories are subdivided, and a definition table of policy tool types suitable for the live streaming e-commerce industry is established. This table fully considers the particularity of the policy of the live streaming e-commerce industry and can be used to effectively analyze the methods used to solve specific problems. In terms of policy effectiveness evaluation, this paper fully draws on the analytic frameworks of other researchers and takes policy goals, policy objects, and policy paths into account. Through the data mining of policy texts, this paper studies secondary variables from these perspectives to construct PMC policy evaluation indicators, which can be used to better analyze the pros, cons, and internal consistency of policy content in the live streaming e-commerce industry from a global perspective. In addition, this paper quantitatively analyzes policy publishers and the characteristics of their cooperation network to effectively analyze the centrality of the policy publishers and the degree of synergy among them, build a cross-departmental policy for the live streaming e-commerce industry, and provide effective suggestions for formulating policies. The specific research framework is shown in Fig 2.

As shown in Fig 2, this paper takes 2016, when the live streaming e-commerce industry began to rise, as the starting point for collecting and counting its related industry policies. In the three-dimensional analysis framework, policy tools are divided into three types: supply-based, demand-based, and environment-based. Sub-categories of policy tools used under these criteria are established to distinguish the specific areas of relevant policy roles. This division is more multi-dimensional and comprehensive, and the operation method is relatively specific, which is more suitable for the relevant policies of the live streaming e-commerce industry in this paper. In the dimension of policy effectiveness evaluation, ROST CM software is used to mine the policy text, select variables through high-frequency word statistics and a keyword semantic network, build the PMC index model of industry-related policies, and construct the PMC surface diagram to intuitively display the pros and cons of the policy. With respect to the dimensions of the policy publishers, this paper uses Ucinet software to conduct a social network analysis of policy publishers in the live streaming e-commerce industry, to study the characteristics of the cooperation network and clarify the centrality and degree of synergy between them.

## 4. Data collection and acquisition

In 2016, the live streaming e-commerce industry grew rapidly due to the epidemic outbreak and Internet development. To ensure the healthy development of this new consumption modality, the government issued many policies. Therefore, this paper started from 2016 to the present, using “live streaming”, “live streaming e-commerce”, “online transaction” and other terms related to the live streaming e-commerce industry as keywords to search for PKULAW.CN.SCHOOL, the Chinese government website, and the official websites of various ministries and commissions. Due to the mixed quality of relevant policies, to ensure the comprehensive, scientific, and representative selection of policies, this paper organizes and screens the policy texts according to the following principles: First, the policy texts at the national level are selected, and the publisher is the central legislature or the State Council and its ministries and commissions, while local policies are not adopted. Second, the policy content is directly related to the live streaming e-commerce industry. Third, the policy types include laws and regulations, official administrative documents, industry standards, and other documents. Fourth, the policies are currently valid. A total of 59 relevant policy texts related to the live streaming e-commerce industry are ultimately collected, as shown in Table 1. Moreover, for convenience, this paper abbreviates the names of the various departments according to the domain names of the official websites of the various departments of the country. The results are shown in Table 2.

**Table 1:** Policy text statistics.

No	Policy name	Issuing time	Implementing time
1	Provisions on the Administration of Internet User Account Information	2022.06.27	2022.08.01
2	Notice of NRTA and MCT on Printing and Distributing the Code of Conduct for Online Live Streaming Host	2022.06.22	2022.06.22
3	Opinions of WENMING, MCT, NRTA, and CAC on Regulating Online Live Rewards and Strengthening the Protection of Minors	2022.05.07	2022.05.07
4	Notice of SAMR on Printing and Distributing the Administrative Measures for the Creation of Online Market Supervision and Service Demonstration Zones (Trial)	2022.04.26	2022.04.28
5	Notice on the Special Project “Rectification of Chaos in the Online Live Streaming and Short Video”	2022.04.15	2022.04.15
…	…	…	…
59	Guiding Opinions of SAIC on Promoting the Healthy Development of Internet Service Transactions and Regulating the Behavior of Internet Service Transactions (Interim)	2016.01.08	2016.01.08

**Table 2:** Abbreviation of department name.

Full name	Abbreviation
National Administration of Traditional Chinese Medicine	NATCM
Agricultural Development Bank Of China	ADBC
China Advertising Association	CAA
China Securities Regulatory Commission	CSRC
China Banking and Insurance Regulatory Commission	CBIRC
The State Council Information Office of the People’s Republic of China	SCIO
Central Commission for Guiding Cultural and Ethical Progress	WENMING
Ministry of Transport of the People’s Republic of China	MOT
The People’s Bank of China	PBC
National Campaign Against Pornography Working Group office	SHDF
General Office of the Standing Committee of the National People’s Congress	GOSCNPC
The National People’s Congress of the People’s Republic of China	NPC
The Ministry of Public Security of the People’s Republic of China	MPS
Ministry of Agriculture and Rural Affairs of the People’s Republic of China	MOA
National Health Commission of the People’s Republic of China	NHC
Ministry of Commerce of the People’s Republic of China	MOFCOM
The State Council of the People’s Republic of China	GOV
Anti monopoly Committee of the State Council	AMCSC
State-owned Assets Supervision and Administration Commission of the State Council	SASAC
Cyberspace Administration of China	CAC
National Development and Reform Commission	NDRC
State Administration for Industry and Commerce of the People’s Republic of China	SAIC
State Administration for Market Regulation	SAMR
National Radio and Television Administration	NRTA
The State Administration of Press, Publication, Radio, Film and Television of the People’s Republic of China	SAPPRFT
State Taxation Administration	CHINATAX
National Medical Products Administration	NMPA
General Administration of Quality Supervision, Inspection and Quarantine of the People’s Republic of China	AQSIQ
China Food and Drug Administration	CFDA
Ministry of Industry and Information Technology of the People’s Republic of China	MIIT
Ministry of Culture and Tourism of the People’s Republic of China	MCT
The Supreme people’s Court of the People’s Republic of China	COURT
National Forestry and Grassland Administration	FORESTRY
General Administration of Customs of the People’s Republic of China	CUSTOMS
China National Intellectual Property Administration	CNIPA
Ministry of Finance of the People’s Republic of China	MOF
State Post Bureau of the People’s Republic of China	SPB

Table 3 shows the monthly change trends and types of policy texts released. The time is subject to the official implementation date of the policy. The type of policy text is determined by the classification of policy documents on the official website of the Central People’s Government of the Communist Party of China, and the policies not clearly divided are set as “other”. Table 3 shows that, in terms of the number of released policies, the number in 2020 was the largest, followed by that in 2016, which was closely related to the actual environment at that time. In 2016, the development of smart phones + 4G led to an explosion of various live streaming platforms, and live streaming e-commerce was the first to attract the attention of the public. This new consumption approach allowed the government and all walks of life to see new economic drivers. Therefore, the government issued many policy measures in that year to create an environment for its development. In 2020, impacted by the epidemic, the development of the live streaming e-commerce industry was objectively further promoted, which gave birth to various forms of industry chaos, and its marketing environment deteriorated rapidly. To regulate industry chaos, the government has issued a great number of normative policies to safeguard the rights and interests of consumers. In terms of policy types, among the 59 industry-related policies, the proportion of notification policy texts is the largest, accounting for 57.63%, indicating that during the development of the industry, the government tends to adopt policies with indicative nature and mandatory arrangement functions. In contrast, the number of other types of policies, such as “announcements” and “opinions”, is relatively small, accounting for 42.37%, which aims to supplement the norms of the live streaming e-commerce industry.

**Table 3:** Monthly change trend and type of released policy texts.

Year	Notification	Opinion	Announcement	Commands	Laws and regulations	Legal	Others	Total	Proportion
2022	4	1	0	1	0	1	0	7	11.86%
2021	5	0	0	0	0	0	2	7	11.86%
2020	8	2	1	3	0	0	2	16	27.12%
2019	5	2	0	0	1	0	0	8	13.56%
2018	7	0	0	1	0	0	0	8	13.56%
2017	1	0	0	0	1	0	1	3	5.08%
2016	4	3	0	2	0	0	1	10	16.95%
Total	34	8	1	7	2	1	6	59	-
Proportion	57.63%	13.56%	1.69%	11.86%	3.39%	1.69%	10.17%	-	-

## 5. Analysis of the three-dimensional framework

### 5.1 Policy tools

#### 5.1.1 Definition of policy tools

According to the research results of domestic and foreign scholars, as well as the basic definition, type and classification, policy tools can be divided into different categories and use types. At present, the classification method of policy tools proposed by Rothwell and Zegveld [17] is recognized; i.e., the basic policy tools are divided into three types: supply-based, environment-based, and demand-based. This paper draws on the above-mentioned policy tool classification method to study the policies of China’s live streaming e-commerce development and takes this as a dimension in the analysis process. Supply-based policy tools are mainly reflected in the driving force of policy development in the live streaming e-commerce industry; these tools include the direct supply of talent, funds, technology, and other elements to ensure resource demand for development. Demand-based policy tools mainly focus on the market, which is reflected in the pulling force of policies on the live streaming e-commerce industry, including government purchases, public services, demonstration promotion, and trade control. Environment-based policy tools, as external factors, indirectly impact the live streaming e-commerce industry, through financial support, tax incentives, standards, and regulations. The above three types of policy tools can be further subdivided according to the content of industry-related policy texts, and their specific classifications and interpretations are shown in Table 4.

**Table 4:** Classification and explanation of policy tools.

type	name	Definition	Example	source
Supply-based type1	Infrastructure construction11	The government establishes or relies on the existing infrastructure to provide platform support for the live streaming e-commerce industry, including information infrastructure, digital construction, logistics platform construction, and information sharing mechanism construction.	Strengthen information network infrastructure building. Further increase new infrastructure buildings such as 5G networks, data centers, industrial Internet, and Internet of Things, and give priority to covering core business districts, key industrial parks, and important transportation hubs…	Opinions of GOV on Leading the Accelerated Development of New Consumption with New Formats and New Models
	Talent training12	Provide talent training for activities related to the live streaming e-commerce industry, and accelerate the development of new economic formats	Carry out training for law enforcement officers and effectively improve the level of law enforcement. Local industry and commerce and market supervision departments should scientifically formulate training plans in light of local conditions, and carry out centralized training for law enforcement officers at all levels of supervision…	Notice of SAIC on Doing a Good Job in the Implementation of the Interim Measures for the Administration of Internet Advertisements
	Element input13	The government provides materials, funds, technology, guidance and other assistance to the live streaming e-commerce industry	Intensify financial support. Increase investment in weak links in the digital economy, break through the shortcomings and bottlenecks restricting the development of the digital economy, and establish a long-term mechanism to promote the development of the digital economy…	Notice of GOV on Printing and Distributing the “14th Five-Year Plan” Digital Economy Development Plan
	Innovation drive14	Innovate the new format of the Internet economy to promote the transformation and upgrading of all walks of life, and contribute to the development of the live streaming e-commerce industry	Vigorously develop “Internet + production”. To meet the needs of industrial upgrading, promote the deep integration of Internet platforms with industrial and agricultural production, improve production technology, improve innovative service capabilities, and vigorously promote the application of materials in the real economy…	Guiding Opinions of GOV on Promoting the Standardized and Healthy Development of the Platform Economy
Demand-based type 2	Poverty alleviation and employment21	Provide safeguard measures activities related to the live streaming e-commerce industry, including targeted poverty alleviation, employment services, and economic development	Serve the overall situation of the country and help poverty reduction. All online audio-visual program service agencies should actively serve the overall strategy of national economic and social development, especially in response to the major deployment of national poverty reduction, through live e-commerce, short-sighted…	Notice of NRTA on Strengthening the Management of Online Audiovisual E-commerce Live Programs and Advertising Programs During the “Double 11” Period
	Foreign exchange22	Conduct scientific research cooperation, information sharing, technical exchanges, etc. with overseas governments and institutions	The state promotes the establishment of exchanges and cooperation with cross-border e-commerce between different countries and regions, participates in the formulation of international rules for e-commerce, and promotes international mutual recognition of e-signatures and e-identities.	Electronic Commerce Law of the People’s Republic of China
	Government purchase23	Products, technologies, services, etc. purchased by the government from the market for the development of the live streaming e-commerce industry	Implement government procurement to support the development of SMB, government entities should reserve more than 30% of the total annual procurement budget of the department for small and medium-sized enterprises, of which the proportion reserved for small and micro enterprises is not less than 30%.	Notice of MIIT, NDRC, MOF, and SASAC on Printing and Distributing the Three-Year Action Plan for Promoting the Inclusive Development of Large, Medium and Small Enterprises
	Public service 24	By providing personalized digital cultural services, improve supply and demand adaptation and public satisfaction, and accelerate economic development	Improve the efficiency of “Internet + Government Services”. Comprehensively improve the functions of the national integrated government service platform, accelerate the standardization and facilitation of government services, and continuously improve the digitalization of government services…	Notice of GOV on Printing and Distributing the “14th Five-Year Plan” Digital Economy Development Plan
	Feedback and complaint 25	Establish efficient and smooth feedback channels to collect public feedback	Improve the mechanism for accepting and handling complaints and reports. Gradually integrate the original industry and commerce, quality inspection, food and drug, price, intellectual property and other complaints and reporting calls and externally set up websites, mobile Apps, WeChat official accounts, WeChat Mini Program and other complaints…	Notice of SAMR on Printing and Distributing the Work Plan for the Governance of Key Areas of Counterfeiting and Shoddy Products (2019–2021)
	Promotion 26	Use various measures to publicize the relevant network knowledge of the development of live streaming, e-commerce and provide public recognition.	Guide guardians of minors to actively learn online knowledge, and strengthen the education, demonstration, guidance and supervision of minors’ online behavior. Support all sectors of society to jointly carry out publicity and education…	Opinions of WENMING, MCT, NRTA, and CAC on Regulating Online Live Rewards and Strengthening the Protection of Minors
	Social participation27	Guide all social forces to participate in activities related to the development of live streaming e-commerce, such as encouraging social supervision, public-private cooperation, social openness, etc	The market supervision and management department guides online transaction operators, online transaction industry organizations, consumer organizations, and consumers to participate in the governance of the online transaction market, and promotes the improvement of diversified participation and effective coordination…	Measures for the Supervision and Administration of Online Transactions
	Pilot demonstration28	Conduct pilot projects and establishes demonstration models to drive the healthy development of the industry	When compiling the work plan for the creation of an e-commerce demonstration city, it is necessary to fully investigate and listen to the opinions of all walks of life; it is necessary to avoid the practice of not paying attention to regulation and policy construction, and only paying attention to the construction of facilities, parks, and engineering projects…	Notice of NDRC and other seven departments on promoting the development of e-commerce
Environment-based type 3	Target planning31	The goals and strategies for the development of the live streaming e-commerce industry formulated by the government, including political position, ideological understanding and work task arrangement, etc.	The overall requirements for rectifying false and illegal advertisements are: adhere to the guidance of Xi Jinping Thought on Socialism with Chinese Characteristics for a New Era, and thoroughly study and implement the 19th CPC National Congress…	Eleven departments including SAMR issued the "Inter-ministerial Alliance for Rectifying False and Illegal Advertisements" The work points of the meeting in 2020 and the inter-ministerial joint meeting on the rectification of false and illegal advertisements Notice of Work System
	Policy support32	The government’s credit support, tax incentives, financial services and other related policy support for enterprises and individuals	Research to further optimize tax collection and management measures for enterprises in the new consumption field, and give better play to the effect of tax reduction and fee reduction policies…	The intention of GOV to lead the accelerated development of new consumption with new business formats and new models
	Market regulation33	The government takes relevant measures to maintain the normal operation of the live streaming e-commerce market, including unfair competition issues, market order, and strengthening market inspections	Actively create a fair competition environment for online live streaming. Platforms and policy publishers are not allowed to make any comments on the main body of commodity production and operation, as well as the performance, function, quality, source, honors and qualifications of commodities…	Notice of CAC, CHINATAX, and SAMR on Issuing the Opinions on Further Regulating the Profitable Behavior of Online Live Broadcasting and Promoting the Healthy Development of the Industry
	Laws regulation 34	Supervise and regulate activities related to the live streaming e-commerce industry	Strike down on tax-related violations and crimes. Investigate and punish tax evasion and other tax-related violations and crimes in accordance with the law, and publicly expose typical cases with serious circumstances, bad nature, and strong social reactions…	CAC, CHINATAX, SAMR The bureau issued the "On Further Regulating the Profitable Behavior of Webcasting and Promoting the Healthy Development of the Industry" Opinions "notice
	Insurance 35	Establish safeguard mechanism such as privacy protection and information security, and urge relevant platforms to strengthen their supervision and establish complaint and feedback channels to protect consumer rights and interests	The government has established safeguards such as privacy protection and information security, and urged relevant platforms to strengthen their own supervision and establish complaint and feedback channels to protect consumer rights and interests	Notice of CAC, MPS, MOFCOM, MCT, CHINATAX, SAMR and NRTA on Printing and Distributing the “Administrative Measures for Online Live Streaming Marketing (Trial)”
	Standard norms 36	Formulate unified industry standards and specifications, promote industry standardization, and ensure the orderly development of the live streaming e-commerce industry	Standardize the application of key functions. Lists and "gifts" are important functional applications that attract young people to “watch” and interact. Platforms should cancel all reward lists within one month of the release of this opinion, and prohibit the use of reward quotas as the sole basis…	Opinions of WENMING, MCT, NRTA, and CAC on Regulating Online Live Rewards and Strengthening the Protection of Minors
	Encouragement and support 37	Encourage and support each platform in the live streaming e-commerce industry to build its regulatory system to strengthen industry self-discipline, and encourage the sharing of relevant information resources between platforms and departments	Encourage the online e-commerce live streaming e-commerce platform to help economic development, improve people’s livelihood, tackle poverty, upgrade industries and connect supply and demand through organizing e-commerce activities	NRTA on Strengthening the Management of Live Streaming of Online Show and e-commerce Live Streaming
	Business coordination 38	The national network information department is responsible for the overall coordination of the national network information content ecological governance and related supervision and management, and the relevant competent departments do a good job in the network information content ecological governance according to their respective responsibilities	Strengthen overall planning and coordination. In accordance with the principle of "who is in charge, who is responsible", we will build a multi field, cross departmental collaborative work pattern to form a strong overall planning, smooth coordination, each performing its own duties, and each bearing its own responsibilities	Regulations on Ecological Governance of Network Information Content

#### 5.1.2 Encoding of policy tools

This paper uses Nvivo 12 qualitative analysis software to encode, classify and quantify the content of policy tools in the policy text. First, policy clauses or chapters are taken as the smallest analysis unit, and the authors subsequently encode them according to the “policy number-point number”. The encoding example is shown in Table 5, and the corresponding reference points are set in Nvivo. Second, the attributes are summarized based on the clause content, and the specific tool name is clarified. Finally, the distribution of nodes is counted to convert the text content into data for quantitative analysis. Moreover, due to the comprehensive content of some policy texts, to ensure accurate classification while performing frequency statistics, it is necessary to repeat the counting according to the type of policy tool. For example, if an analysis unit involves regulatory control and business coordination simultaneously, each of the two policy sub-tools is counted once.

**Table 5:** Classification and explanation of policy tools.

No	Name	Text analysis unit	Encoding	Policy tool
1	Provisions on the Administration of Internet User Account Information	Clause 1 These Provisions are formulated in order to strengthen the management of Internet user account information. Promote the core socialist values, and safeguard national security.	1–1	31
		Clause 2 Internet users registering and using Internet users at Internet information service providers within the territory of the People’s Republic Of China… These Provisions shall apply.	1–2	37
		Clause 3 The national network information department shall be responsible for the supervision and management of the national Internet user account information. Local network information departments shall supervise and manage the work according to their responsibilities.	1–3	34

#### 5.1.3 Analysis of policy tools

After the policy tool is encoded and the composite policy tool is added, a total of 2266 policy tools remain and the results are shown in Table 6.

**Table 6:** Distribution of policy tools.

Policy tool	Category	Response time	Share ratio	Total	Total proportion
1 supply-based	Infrastructure construction11	111	32.94%	337	16.77%
	Talent training 12	27	8.01%		
	Element input13	74	21.96%		
	Innovation drive14	125	37.09%		
2 demand-based	Poverty alleviation and employment 21	20	4.91%	407	20.26%
	Foreign exchange 22	13	3.19%		
	Government purchase23	1	0.25%		
	Public service24	45	11.06%		
	Feedback and complaints 25	86	21.13%		
	Publicity and promotion 26	52	12.78%		
	Social participation 27	159	39.07%		
	Pilot demonstration 28	31	7.62%		
3 environment-based	Goal plamning31	105	8.30%	1265	62.97%
	Policy support 32	48	3.79%		
	Market regulation 33	76	6.01%		
	Regulatory control 34	336	26.56%		
	Safeguards 35	167	13.20%		
	Standard specification 36	352	27.83%		
	Encouragement and support 37	96	7.59%		
	Business collaboration 38	85	6.72%		

Overall, the relevant policies of China’s live streaming e-commerce industry use policy tools more comprehensively and take supply-based, environment-based, and demand-based policy tools into account, but the differences among different types of policy tools are significant. Among them, environment-based policy tools are used the most frequently, with 1,265 items, accounting for 62.97%. This finding indicates the government prefers to regulate the macro environment through indirect means to ensure the orderly development of the live streaming e-commerce industry. Demand-based policy tools are used in second place in terms of frequency, accounting for 20.26%. Supply-based policy tools are used in the least order, accounting for 16.77%. Compared with those of environment-based policies, there are fewer other two types of policies, which concentrate on the early stage of the development of the live streaming e-commerce industry, i.e., 2016–2018. The proportions of sub-categories of tools used for the three types of policies also significantly differ. Among them, within the supply-based policy tools, innovation drive and infrastructure construction play important roles in the policy content of these tools, accounting for 37.09% and 32.94%, respectively. The frequency of talent training and element input is significantly lower, especially talent training, which is only 8.01%. This shows that the government has invested far less in capital, materials, and talent in the live streaming e-commerce industry. Among the demand-based policy tools, social participation and feedback complaint tools account for a relatively high proportion of the total, but other policy tools covering content such as poverty alleviation and employment, government purchases, publicity and promotion are significantly lacking, indicating that the government’s driving force for live streaming e-commerce is not enough. In addition, within environment-based policy tools, standards and norms (27.73%) as well as regulations and controls (26.56%) are the government’s most commonly used regulatory means. However, the use of other policy tools, such as target planning, policy support, and market environmental control, is still insufficient, within 10%. It can be seen that government departments have focused their attention on rectifying the behavior of the industry, while policies related to its development, such as target norms and encouragement and support, have not been given great attention, as they have curbed the enthusiasm of the economic market to a certain extent.

In summary, although the current live streaming e-commerce industry has developed quickly, its policy has a structural imbalance of overflowing environment-based policy tools and weakening demand-based and supply-based policy tools. This weakens the government’s role in promoting market demand, which is not conducive to the advantages of the live streaming e-commerce industry in driving economic recovery affected by the epidemic. Therefore, with the further development of the live streaming e-commerce industry, policy system optimization should focus on targeted and scientific policy tools. The government should avoid the single use of policy tools and appropriately reduce the use of environment-based policy tools, coordinate supply-based policy tools, and expand demand-based policy tools, thus strengthening the advantages of the live streaming e-commerce industry in poverty alleviation, flexible employment, cross-border e-commerce, and economic transformation. On the other hand, the government should enhance the proportional coordination of sub-category tools within each policy tool. In particular, first, in addition to strengthening the four elements of infrastructure, technology, talent, and capital, timely insights into the new needs of information security subjects should be gained, and consider various supportive measures such as public services and tax incentives should be considered. Second, it is necessary to intensify publicity, promote cooperation between the government and external enterprises, value resource sharing and network attack and defense simultaneously, and promote the benign development of the market. Finally, strategies are needed to enhance compulsory industry norms, upgrade industry normative entries into mandatory laws and regulations, protect the interests of all parties, and enable healthy development in the live streaming e-commerce industry.

### 5.2 Policy effectiveness evaluation

Policy effectiveness evaluation estimates and evaluates the content, implementation, goal achievement, and other effects of a policy. The policy is measured and analyzed based on a series of specific evaluation criteria and research methods to evaluate the effect, impact, and value of policy implementation. Policy effectiveness evaluation directly contributes to policy formulation, implementation, and feedback adjustment. At present, there is a certain degree of subjective tendency and ambiguity in the research on policy effectiveness evaluation, and the PMC index model is a quantitative policy evaluation analysis method. The innovation of this index model is the use of binary digits 0 and 1 to balance each variable, and it emphasizes that the number and weight of variables should not be limited. Based on this, the PMC index model can analyze the internal heterogeneity and pros and cons of a policy from a multi-dimensional perspective, and visually display the advantages and disadvantages of each dimension of the policy through the PMC surface diagram. At present, the PMC index model is widely used in the quantitative analysis of policy texts in many fields. The typical applications of these methods are as follows: Zhang and Yang [18] used the PMC to construct a policy text evaluation model, and propose three optimization strategies for the education policy of border ethnic minorities. Liu and Li [19] applied the PMC to evaluate the effectiveness of the 20 policies for promoting the employment of college students from 2015 to 2020. The results showed that 19 of the 20 policies were acceptable or excellent, accounting for 95% of the total publications, indicating that most of the current policies could effectively promote the employment of college students. However, representative policies could be optimized and upgraded according to two different ideas: “policy tendency-policy field-target-policy focus” and “policy tendency-policy field-policy effectiveness-policy focus-target-policy nature”. Therefore, to comprehensively evaluate the effectiveness of the current policies related to the live streaming e-commerce industry, based on the relevant literature, this paper further extracts evaluation indicators suitable for the policies of the live streaming e-commerce industry by mining the policy texts and constructing the PMC index model. According to the PMC index scores, the pros and cons of the relevant policies of the live streaming e-commerce industry are identified, and suggestions for policy optimization are put forward to enhance the resilience of the policy system of the live streaming e-commerce industry.

#### 5.2.1 Construction of the policy PMC index model

**Fig 3:**
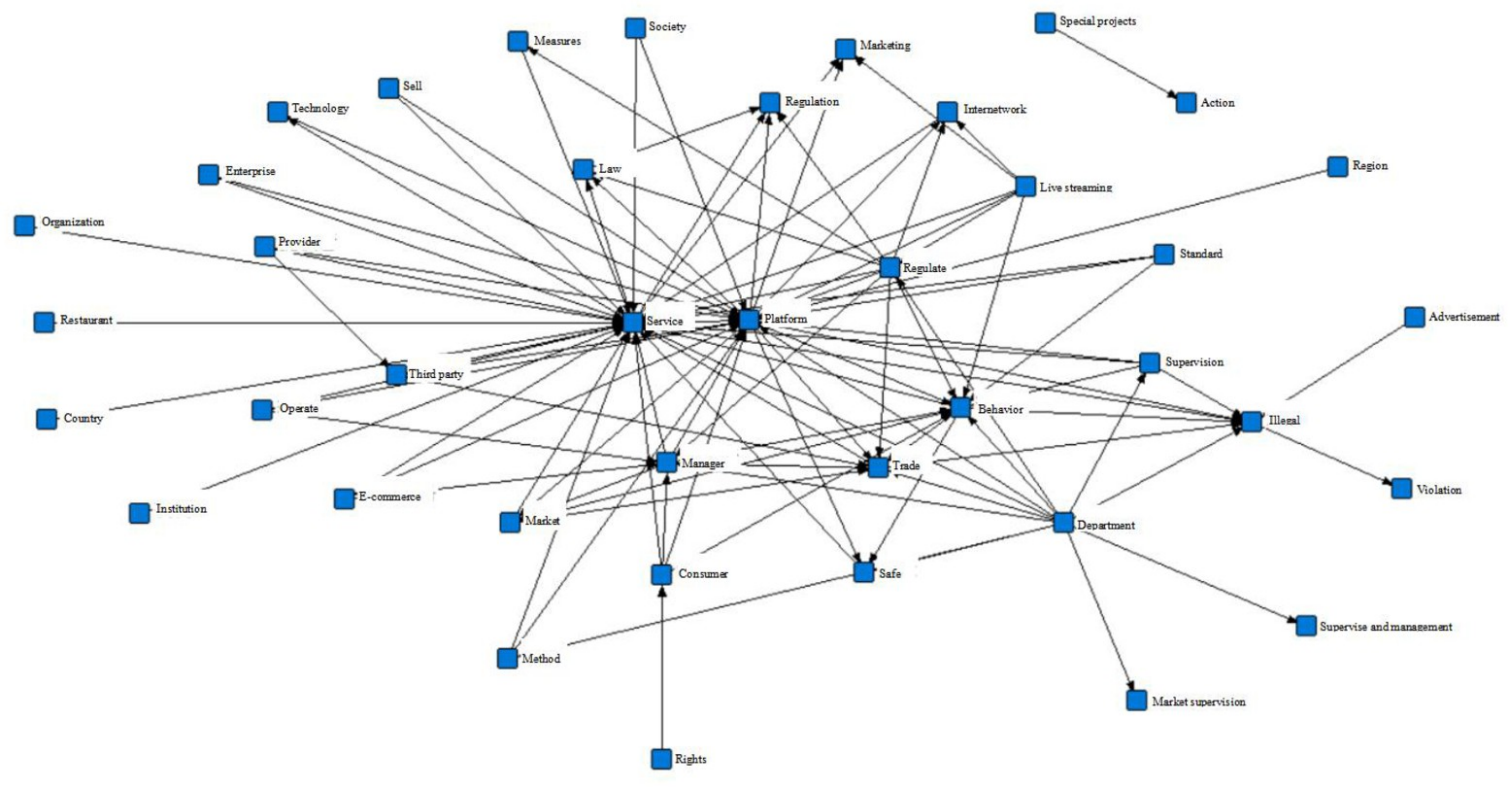
Semantic network of subject words.

Variable classification and parameter identification are the most critical steps before evaluating policy effectiveness. The first-level variables include a total of 9 first-level indicators reflecting the nature of the policy and the timeliness of the policy based on the studies of scholars such as Estrada [20], Cai et al. [21], and Wang et al. [22]. Second, to enhance the adequacy of variable parameters, in addition to referring to the above-mentioned scholars, this paper mines relevant policy texts in the live streaming industry through data mining to optimize the variables and parameters in the PMC index model. The specific process is as follows:

First, the ROST CM software is used to import the collected policy texts into the system for word frequency statistics, and data cleaning is performed on high-frequency words, i.e., to delete words such as “strengthen”, “unit”, “province”, “strengthen”, “speed up” and other level words; modifiers; and irrelevant words; furthermore to merge keywords with similar semantics and consistent descriptions, such as “fine” into “punishment”, “competent department”, “regulatory department”, “State Council”, etc., into “relevant departments”, “information services”, “network services”, “public services”, etc., into “relevant departments”, “service system”, etc., and then to select the first 150 words to form the final high-frequency vocabulary. The results are shown in Table 7. Finally, the topic word semantic network map is constructed through the high-frequency vocabulary, as shown in Fig 3. The operations reveal the specific characteristics of the policy content, paving the way for variable classification and parameter setting.

**Table 7:** Distribution of high-frequency words in policy texts.

Vocabulary	Frequency	Vocabulary	Frequency	Vocabulary	Frequency	Vocabulary	Frequency	Vocabulary	Frequency
Internet	1657	Account	236	Special projects	125	Reform	76	Government	41
Supervision	1445	Society	226	SMB	125	Informatization	73	Person in charge	40
Platform	1424	Punishment	205	Management	123	Standard	72	Prevention	38
Services system	1294	Region	202	Synergy	121	Ecology	71	Appointment	38
Department	1193	Mechanism	198	Website	119	Evaluation	71	Verify	37
Information	1152	Digitalization	195	Supervise	118	Quality	69	Arrange	37
Manager	754	Publicity	190	Govern	114	Return	64	Fair	37
Internet work	596	Public	188	Propagate	112	False and shoddy	64	Merchant	37
E-commerce	570	Industry	185	Training	110	Save	63	Integrity	36
Illegal	559	New format	183	Risk	107	Tip	62	Supply	35
Regulation	559	Institution	180	Business	107	Explore	62	Entrepreneurship	35
Market	555	Credit	177	Guide	104	Logistics	62	Spot check	35
Trade	523	Product	171	Complaint	102	Poverty alleviation	61	Commercial	34
Development	516	Cooperation	168	Industrial	102	Policy	59	Upgrade	34
Advertisement law	472	False	160	Responsible	101	Fair competition	59	Submitted	34
Consumer	398	Law	157	Maintenance	96	Permission license	53	Jurisdiction	34
Enterprise	368	System	154	Lead	93	Spread	52	resource integration	34
Online live streaming	361	Encourage	153	Live streaming room	92	Transform	52	Coordinate	33
Protection	343	Network information	148	Environment	90	Cross-border	51	Capital	33
Responsibility	340	Consumption	145	Antitrust	90	Principle	50	Invest	33
Legal rights	320	Review	142	Agreement	88	Marketing	50	Management institution	32
Operate	312	Law enforcement	141	Concert	84	Publisher	50	Warn	32
Services provider	310	Culture	141	Competition	84	Operation	48	Element	32
Innovation	284	Obligation	140	Infrastructure	80	Untrustworthy	48	Tourism	31
Sell	273	Share	133	To urge	79	Strike hard	46	Usher	31
Live streaming marketing	270	Host	132	Resource	79	Self-discipline	44	Open-up	31
Economy	259	Record	130	Order	79	Handle	43	Supply chain	30
Activity	259	Check	128	Minor	78	Channel	43	Media	30
Cyber security	256	Report	127	Finance	78	Assist	43	Blacklist	29
Investigation	241	Operator	126	Demonstrate	77	Warning	42	Test	26

As shown in Table 7 and Fig 3, industry regulations, public services, business entities, relevant departments, and other nodes occupy central areas, connect with other keywords mostly, and represent the core topic words of policy texts, indicating that live streaming e-commerce policies surround these aspects. According to the distribution of high-frequency words, the content of the policy text is as follows: (1) the government intensifies the supervision of various platforms, merchants, and live streaming hosts by continuously improving the industry supervision system and strengthening law enforcement; (2) relevant industry norms are gradually improved, and market orders are improved; (3) keywords such as digitization, innovation, transformation and upgrading, and new business formats reflect that the formulation of the current live streaming e-commerce policies also pays attention to technological innovation and development and application of the Internet; and (4) topics such as sharing and resource collaboration reflect that while developing live streaming e-commerce, the management of data sharing and resource optimization are attached to great importance. Therefore, in this paper, combined with the settings of variable parameters in the previous relevant literature, 9 first-level variables are adjusted and established, and several second-level variables are set. The results are shown in Table 8.

**Table 8:** Policy evaluation indicators and secondary variables.

First-level variables	Second-level variables	Secondary variable evaluation criteria	Source or basis
Policy nature X1	X1:1 predict	Whether it is predictive	Estrada, 2011 [20]
	X1:2 regulate	Whether it is regulatory	
	X1:3 advise	Whether to make recommendations	
	X1:4 support	Whether to provide support	
	X1:5 guide	Whether it is guided	
Policy timeliness X2	X2:1 long-term	Whether the policy involves more than 3 years of content	Estrada, 2011 [20]
	X2:2 middle-term	Whether the policy involves 1~3 years of content	
	X2:3 short-term	Whether the policy involves less than 1 year of content	
Policy perspective X3	X3:1 Macro	Whether it contains macro level	Cai et al. 2021 [21]
	X3:2 Micro	Whether it contains micro level	
Policy evaluation X4	X4:1 Clear goals	Whether the policy goal is clear	Cai et al. 2021 [21]
	X4:2 Sufficient basis	Whether the policy basis is sufficient	
	X4:3 Detailed plan	Whether the policy plan is detailed	
	X4:4 reasonable plan	Whether the policy plan is reasonable	
Policy region X5	X5:1 politic	Whether it involves the political field	Mining and self-building according to policy text
	X5:2 economy	Whether it involves the economic field	
	X5:3 social service	Whether it involves the social service field	
	X5:4 innovate	Whether it involves the innovation field	
	X5:5 technology	Whether it involves the government technology field	
	X5:6market environment	Whether it involves the market environment field	
Policy audience X6	X6:1 government department	Whether it involves the government department field	Mining and self-building according to policy text
	X6:2 enterprise	Whether it involves the enterprise field	
	X6:3 non-profit organization	Whether it involves the non-profit organization field	
	X6:4 public	Whether it involves the public field	
	X6:5 live streaming platform	Whether it involves the live streaming platform	
	X6:6 host	Whether it involves host	
	X6:7 Self-media	Whether it involves the self-media	
Policy support X7	X7:1 government subsidy	Whether it involves the government subsidy	Mining and self-building according to policy text
	X7:2 technology support	Whether it involves the technology support	
	X7:3 information sharing	Whether it involves the information sharing	
	X7:4 talent incentive	Whether it involves the talent incentive	
	X7:5 platform construction	Whether it involves the platform construction	
	X7:6 tax preferential	Whether it involves the tax preferential	
	X7:7 pilot construction	Whether it involves the pilot construction	
	X7:8 publicity and education	Whether it involves the publicity and education	
	X7:9 other support	Whether it involves other support	
Policy priority X8	X8:1 social supervision	Whether it involves the social supervision	Mining and self-building according to policy text
	X8:2 industry regulation	Whether it involves the industry regulation	
	X8:3 government supervision	Whether it involves the government supervision	
	X8:4 network information security	Whether it involves the network information security	
	X8:5 transformation and upgrading	Whether it involves the transformation and upgrading	
	X8:6 infrastructure construction	Whether it involves the infrastructure construction	
	X8:7 market competition	Whether it involves the market competition	
	X8:8 cross-border development	Whether it involves the cross-border development	
	X8:9 poverty alleviation and employment	Whether it involves the poverty alleviation and employment	
Action mode X9	X9:1 mandatory-based	Whether it is mandatory-based	Wang et al. 2019 [22]
	X9:2 service-based	Whether it is service-based	
	X9:3 incentive-based	Whether it is incentive-based	

#### 5.2.2 Construction of a multi-input-output table

The multi-input-output table of the PMC index model is a data analytic framework formed by measuring variables from multiple dimensions, consisting of first-level and second-level variables. In the PMC index model, the weights of all the second-level variables are equal, and the parameter values are binary. If a policy text contains the content of the corresponding second-level variable, the parameter value of the second-level variable is marked as 1; otherwise, it is 0. The analysis results in section 5.1.3 indicate that most policy tools are related to industry norms and supervision. To make a better assessment, this paper selects 5 comprehensive and representative policies. As the research object, a policy effectiveness analysis is carried out. The specific content is shown in Table 9, and the multi-input-output table obtained is shown in Table 10.

**Table 9:** Representative policies related to the development of the live streaming e-commerce industry.

No.	Policy name	Implementation time
P1	Notice of NDRC and other seven departments on promoting the development of e-commerce	2016.05.20
P2	Guiding Opinions of GOV on Promoting the Standardized and Healthy Development of the Platform Economy	2019.08.01
P3	Suggestion of GOV to accelerate development of new consumption with new business formats and new models	2020.09.16
P4	Notice of CAC, MPS, MOFCOM, MCT, CHINATAX, SAMR and NRTA on Printing and Distributing the “Administrative Measures for Online Live Streaming Marketing (Trial)”	2021.05.25
P5	CAC, CHINATAX, SAMR, Notice of “On Further Regulating the Profitable Behavior of Webcasting and Promoting the Healthy Development of the Industry”	2022.03.25

**Table 10:** Multi-input-output table of policy.

First-level variables	Second-level variables	P1	P2	P3	P4	P5
X1	X1:1	1	1	1	0	1
	X1:2	0	1	1	1	1
	X1:3	1	1	1	1	1
	X1:4	1	1	1	0	0
	X1:5	1	1	1	0	1
X2	X2:1	0	0	1	0	0
	X2:2	1	0	0	0	0
	X2:3	1	1	0	1	1
X3	X3:1	1	1	1	0	1
	X3:2	1	1	1	1	1
X4	X4:1	1	1	1	1	1
	X4:2	1	1	1	1	1
	X4:3	1	1	1	1	1
	X4:4	1	1	1	1	1
X5	X5:1	0	1	1	1	1
	X5:2	1	1	1	1	1
	X5:3	1	1	1	0	0
	X5:4	1	1	1	0	0
	X5:5	1	1	1	0	0
	X5:6	1	1	1	0	1
X6	X6:1	1	1	1	1	1
	X6:2	1	1	1	1	1
	X6:3	0	1	1	1	0
	X6:4	0	1	1	1	0
	X6:5	1	1	1	1	1
	X6:6	0	0	0	1	1
	X6:7	0	0	0	0	1
X7	X7:1	0	0	1	0	0
	X7:2	1	1	1	0	0
	X7:3	1	1	1	1	1
	X7:4	0	1	1	1	0
	X7:5	1	1	1	1	1
	X7:6	0	1	1	0	0
	X7:7	1	0	1	0	0
	X7:8	0	0	1	0	1
	X7:9	1	1	1	0	1
X8	X8:1	0	1	1	0	1
	X8:2	1	1	1	1	1
	X8:3	0	1	1	1	1
	X8:4	1	1	1	1	1
	X8:5	1	1	1	0	0
	X8:6	1	1	1	0	0
	X8:7	0	1	0	1	1
	X8:8	1	1	1	0	0
	X8:9	0	1	1	0	0
X9	X9:1	0	0	0	1	1
	X9:2	1	1	1	0	0
	X9:3	1	1	1	0	1

#### 5.2.3 Calculation of the PMC index

The calculation of the PMC index refers to the calculation method of Estrada [23]. The specific steps are as follows:

1. Establish first-level and second-level variables according to the relevant policy content of live streaming.
2. Construct a multi-input-output table and assign specific values to the second-level variables through text mining and formulas (1) and (2).
X~N[0,1] (1)
$$X=\left\{XR:[0\sim 1]\right\}$$ (2)
3. According to formula (3) and the assignment of the second-level variables, calculate the values of the first-level variables.
$$X_{t}\left(\sum_{j=1}^{n}\frac{X_{ij}}{T\left(X_{ij}\right)}\right),\;t=1,2,3,\cdots$$ (3)
  Where *t* is a first-level variable and *j* is a second-level variable.
4. The PMC index of the policy to be evaluated is calculated by summing up formula (4).
$$\begin{array}{l} PMC=\left[X_{1}\left(\sum_{j=1}^{5}\frac{X_{1j}}{5}\right)\right.+X_{2}\left(\sum_{j=1}^{3}\frac{X_{2j}}{3}\right)+X_{3}\left(\sum_{j=1}^{2}\frac{X_{3j}}{2}\right)\\ +X_{4}\left(\sum_{j=1}^{4}\frac{X_{4j}}{4}\right)+X_{5}\left(\sum_{j=1}^{6}\frac{X_{5j}}{6}\right)+X_{6}\left(\sum_{j=1}^{7}\frac{X_{6j}}{7}\right)\\ +X_{7}\left(\sum_{j=1}^{9}\frac{X_{7j}}{9}\right)+X_{8}\left(\sum_{j=1}^{9}\frac{X_{8j}}{9}\right)\left.+X_{9}\left(\sum_{j=1}^{3}\frac{X_{9j}}{3}\right)\right] \end{array}$$ (4)

The PMC indices of various policies related to live streaming e-commerce are obtained. Finally, according to the PMC index value, the consistency of various policies related to live streaming e-commerce is evaluated. There are 9 first-level indicators in total, so the total score of the indicators for each policy is 9. Moreover, according to the perspective of Ruize [24], the scores are rated according to the policy scoring shown in Table 11.

**Table 11:** Ratings of policy scores.

Score	Evaluation
0~5.39	Bad
5.4~6.29	General
6.3~7.19	Good
7.2~9	Excellent

#### 5.2.4 Results and analysis of the PMC index model

The policy evaluation results obtained according to the PMC index model are shown in Table 12. The sag index is 9-PMC, which is used to indicate the degree of difference between the policy to be evaluated and the “perfect policy”.

**Table 12:** PMC index of policy.

No.	X1	X2	X3	X4	X5	X6	X7	X8	X9	PMC index	Sag index	Rank	Degree
P1	0.80	0.67	1.00	1.00	0.83	0.43	0.56	0.56	0.67	6.51	2.49	3	Good
P2	1.00	0.33	1.00	1.00	1.00	0.71	0.67	1.00	0.67	7.38	1.62	2	Excellent
P3	1.00	0.33	1.00	1.00	1.00	0.71	1.00	0.89	0.67	7.60	1.40	1	Excellent
P4	0.40	0.33	0.50	1.00	0.33	0.86	0.33	0.44	0.33	4.53	4.47	5	Bad
P5	0.80	0.33	1.00	1.00	0.50	0.71	0.44	0.56	0.67	6.01	2.99	4	General
Average	0.8	0.4	0.9	1	0.73	0.69	0.6	0.69	0.6	6.41	2.59	-	-

As shown in Table 12, among the five selected live streaming e-commerce policies, the results of two policy evaluations are excellent, one is good, one is medium, and one is bad. The evaluation results of the policies are within an acceptable range. These live streaming e-commerce policies are scientific and feasible to a certain extent, and the synergy of relevant policies is high and can combine the needs of industry and social development. However, some policies also have certain defects and need to be further improved. The average score of each variable shows that, first, policy timeliness has the lowest score, indicating that the relevant policies are not clear enough in long-, medium-, or short-term planning, and that the development goals are relatively simple. Second, policy support and action methods have low scores. Combined with the analysis results of the policy tool dimension in Section 5.1, most of the policies currently released are similar to the selected policies P4 and P5; that is, they are related to industry norms, which shows that the government attaches great importance to industry regulation rather than to promotion. The policy method will inevitably be too limited, the scope of action will be narrow, and the enthusiasm of each subject engaging in live streaming e-commerce will be reduced.

To more clearly observe the results of the quantitative evaluation of various policies in the PMC index model, this paper introduces 9 first-level variables into formula (5) to construct a 3×3 surface matrix, draws a surface map of each policy, and examines the internal consistency level and the effectiveness of the policy structure.


$$\text{PMC}\left(\text{surface}\right)=\left[\begin{array}{ccc} X_{1} & X_{2} & X_{3}\\ X_{4} & X_{5} & X_{6}\\ X_{7} & X_{8} & X_{9} \end{array}\right]$$ (5)

**Fig 4:**
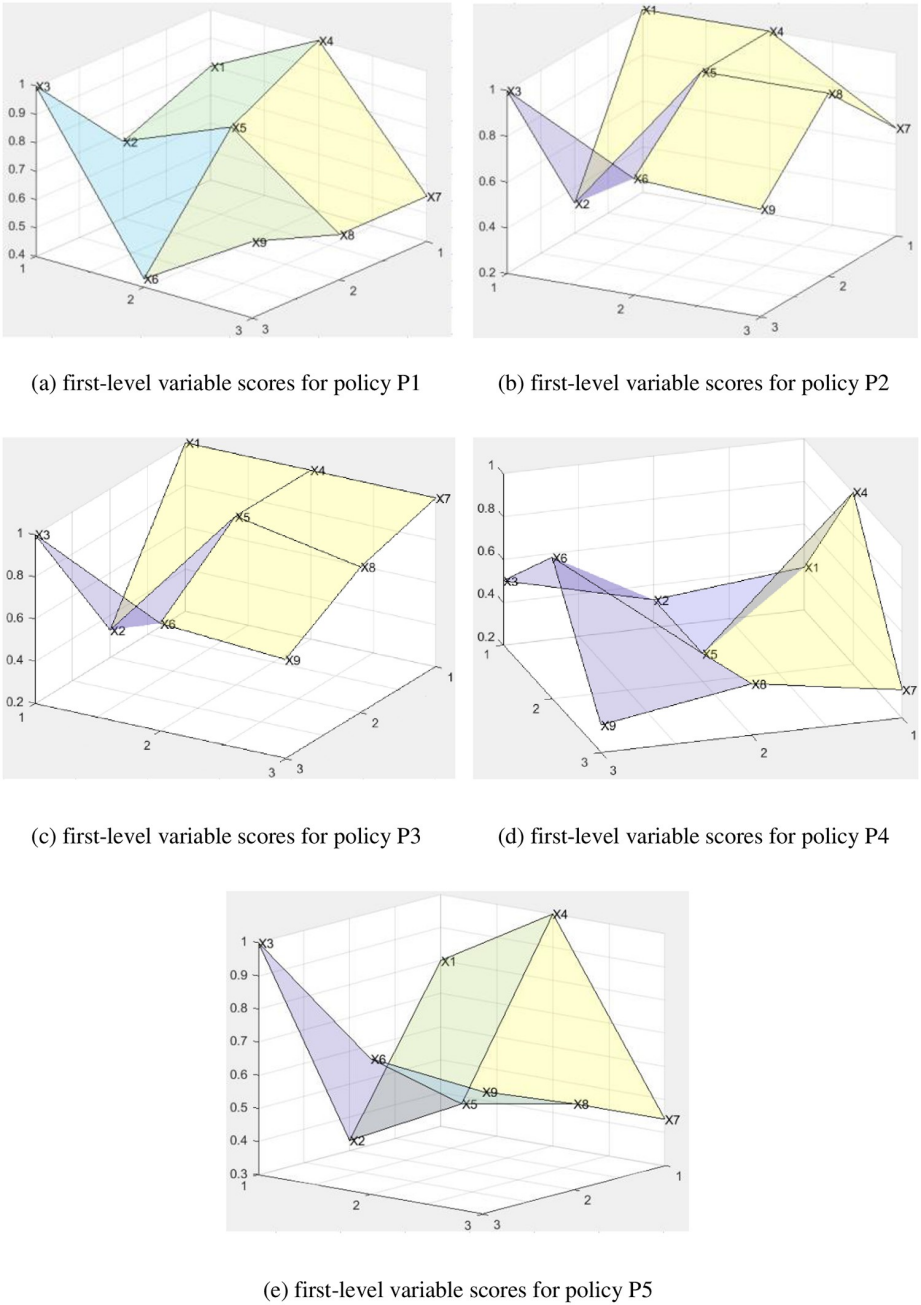
PMC three-dimensional graphs of policy.

The results are shown in Fig 4, where the *x* and *y* coordinate systems correspond to the positions of the first-level variables X1-X9, the *z*-axis corresponds to the score of each first-level variable, and the three-dimensional graphs of P1, P2, P3, P4, and P5 correspond to the positions of *a*, *b*, *c*, *d*, and *e*, respectively.

Fig 4 shows that P2 and P3, which are at the excellent level according to the three-dimensional map of the PMC index of the five policies in Fig 4, are comprehensive policies related to promoting the development of the live streaming e-commerce industry. These policies take general requirements and specific measures into account, involving politics, economics, innovation, services, and other policy areas. They attract a wide audience, including government departments at all levels, businesses, non-profit organizations, citizens, etc., and the consideration of the content of each evaluation index is also relatively balanced. However, there are obvious defects in the timeliness of these policies; for example, the combination of long-term, medium-term and short-term goals is ignored, and the specific timing of medium- and long-term development plans is not given, which is also a common problem of the five selected policies. P1 is also a comprehensive policy on the development of the industry and is released in the initial stage of the live streaming e-commerce industry. Therefore, the understanding of the development trend of the industry is insufficient, resulting in additional gaps in the three aspects of policy audience, policy support, and policy priority. P4 and P5 are policies related to industry norms. Fig 4 shows that, compared with other policies, they are the norm of subject behavior in the industry; thus, except for the policy receptors, the other indicators are very low, indicating that the content of their policies is too limited. However, over-emphasis on regulatory norms for tends to inhibit industry development. Therefore, relevant normative policies should also include corresponding incentives while regulating the industry, and appropriate support should be given to those law-abiding businesses and enterprises in terms of capital, taxation, and technology.

In summary, the relevant policies of China’s live streaming e-commerce industry generally have the following problems: single policy effectiveness; undetailed formulation of long, medium, and short-term goals and a disunited relationship between goals and policy tools. The settings of policy priorities, incentives, and modes of action are not perfect enough. The text content of the policy shows that the policies with lower PMC scores are normative policies, which account for the majority of the policies issued by the government. The current policy content is simple, which results in low policy timeliness, function, and action method scores. Therefore, while ensuring industry norms, the government should also improve the policy paths represented by these variables as follows: First, based on national conditions and the specific live streaming e-commerce situation, the government should formulate small goals with strong operability in stages under the guidance of the overall goal, take the macro- and micro-levels of the policy into account, and improve the operability and effect of the live streaming e-commerce policies. Second, the government needs to enrich its incentive measures in policy content and fully consider the means of government financial subsidies, technical support, tax incentives, and talent incentives to accelerate the development of the live streaming e-commerce industry. Third, to intensify policy publicity, the government can first pilot policy implementation demonstrations in regions and industries where the live streaming e-commerce industry is more mature; then it can gradually expand its application fields, such as medical care, education, tourism, and other fields; and attach importance to accelerating economic transformation and upgrading throughout all walks of life. Fourth, based on standardization and supervision, the government can appropriately strengthen services and incentives such as data sharing and public-private cooperation to prevent excessive inhibition of live streaming e-commerce development.

### 5.3 Policy publishers

The main body of policy publishers refers to the institution or organization that releases relevant policy documents in accordance with legal authority and procedures, which is the core part of the policy system. Schneider and Ingramtt [25] proposed that the formulation and implementation of policies were mainly jointly realized by the macro-control behavior of policy publishers, the selection and application of policy tools and the selection of policy objects. The publisher has significant power in participating in or influencing policy formulation, implementation, supervision, and evaluation. Accordingly, this paper draws on the research methods of policy analysis of Liu et al. [26], and uses Ucinet software [27] to quantitatively analyze the relevant policy publishers and their cooperation network characteristics in the live streaming e-commerce industry through social network analysis and characterization of undirected binary networks to clarify the centrality of the policy publishers and the degree of coordination among the subjects.

#### 5.3.1 Social network analysis

Social network analysis quantitatively combines graph theory and mathematical models to study social actors and their relationships with each other. It investigates the social structure of relationships within a network and the impact of this structure on individual actors or groups. This method has been implemented in a variety of ways in different fields, including business, communication, information science, economics, physics, and biology.

A social network is a collection of social actors as nodes and their relationships with different properties and measures; these can be described as structural, rational, or functional properties. The structural properties depend on the relationships among nodes. It includes the density and size of social networks. Rational properties are connections among nodes and can be described by strength, reciprocity, and multiplicity. Functional properties are transactional meanings, such as how two participants deal with each other. Based on these attributes and metrics, this paper conducts a quantitative analysis of the policy publishers and their relationships with each other. First, the joint release of relevant policies in the live streaming e-commerce industry across multi-department is regarded as cooperation among the subjects, and the cooperation matrix of the policy publishers is determined. Second, the cooperation network diagram is drawn with the help of Ucinet software. Finally, the cooperation matrix is transformed into an undirected binary matrix through the software to calculate the overall network characteristics and node structure characteristics among the subjects. The social network measurement indicators and interpretations are shown in Table 13.

**Table 13:** Social network measurement indicators and interpretations.

Dimension	Indicator	Interpretation
Overall network characteristics	Density	Reflects the average level of interaction between subjects within the overall network
	Average Distance	Reflects the effectiveness of information transfer in the overall network
	Compactness	Reflects the compactness of the overall network (the range is from 0 to 1, the larger value represents the stronger compactness)
Node structure characteristics	Degree	Reflects the importance of nodes in the network, i.e., the key organization
	Between	Reflects the control ability and adjustment ability of the node to other nodes
	Closeness	Reflects the distance between a node and other nodes in the network or the speed at which the overall utility of the node is achieved
	Eigenvector	Reflects the network level depth and location advantages of nodes

#### 5.3.2 Model results

**Fig 5:**
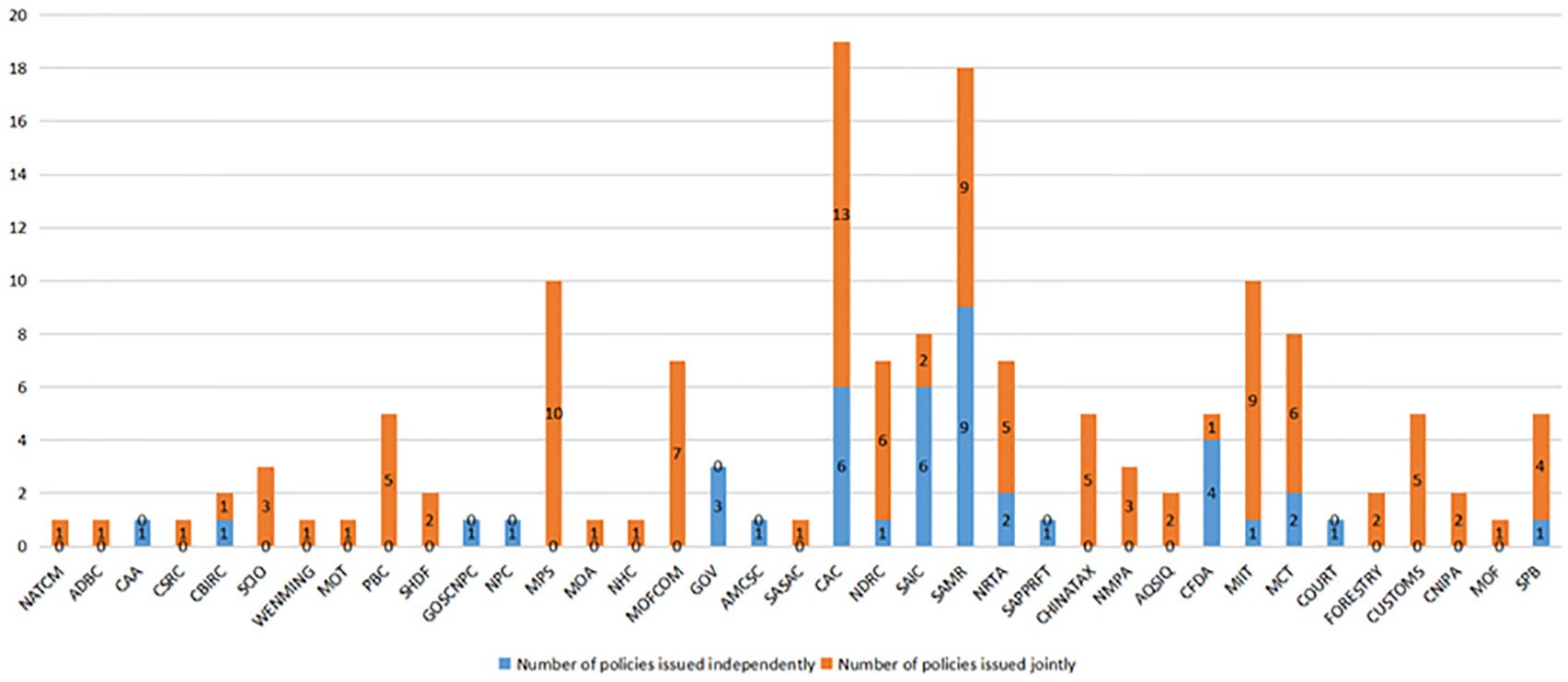
Number and classification of released policies.

A total of 37 policy publishers related to the live streaming e-commerce industry and the release policy situation are shown in Fig 5. The CAC and SAMR publish the largest number of policies, with 19 and 18 issued, respectively, and their joint policy releases also accounted for more than 50% of the total issued. Following MPS and MIIT, the total number of releases reached 10. In general, the majority of joint release policies were implemented, and most of these policies were released after 2018, indicating that the formulation and implementation of relevant policies in the live streaming e-commerce industry have gradually changed from an independent decision-making model to an interconnection model.

**Fig 6:**
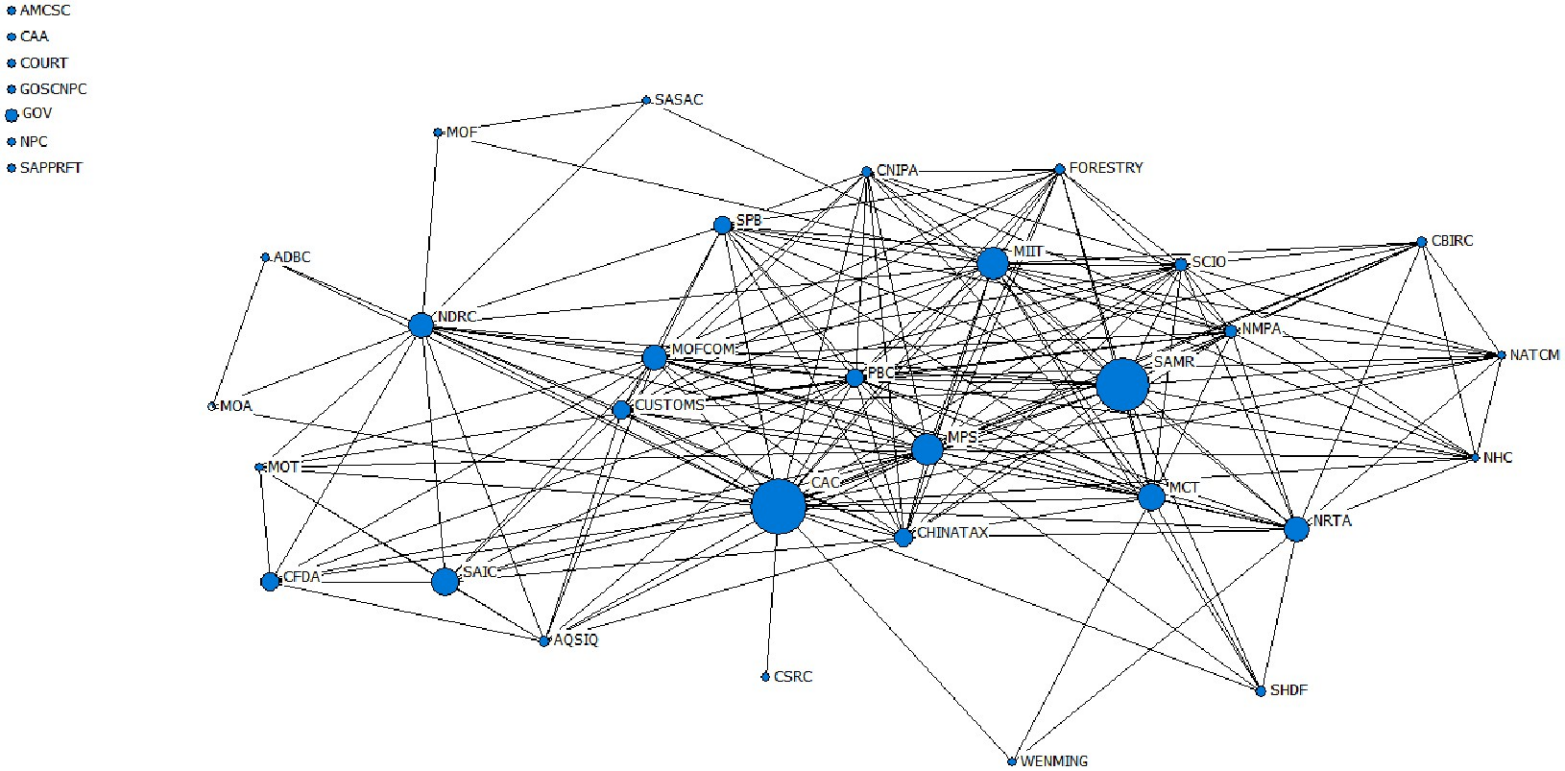
Social network analysis model of policy publishers.

The social network analysis model of policy publishers constructed with Ucinet software, as shown in Fig 6, includes a cooperative relationship with 30 participants, and 7 participants worked independently during this period.

By Ucinet software, According to the output results, the network density in the social network model of publishers is 0.281, the standard deviation of the density is 0.449, the average distance is 1.579, and the cohesion index is 0.466. The data show that the overall structure of the social network of current policy publishers is not close enough, and their relationships are loose. However, the value of the standard deviation is greater than the network density. The cooperation density among some subjects in the network is greater than the overall network density, and there is a local “aggregation” phenomenon. To further analyze their connections, it is necessary to combine the characteristics of the node structure in the social network model. The results are shown in Table 14.

**Table 14:** Centrality of the policy publishers.

Policy publisher	Degree	Between	Closeness	Eigenvector
CAC	75.000	95.216	12.414	0.285
MPS	63.889	20.716	12.245	0.277
PBC	61.111	16.233	12.203	0.272
MIIT	58.333	37.521	12.162	0.253
MOFCOM	52.778	8.794	12.081	0.246
SAMR	52.778	7.021	12.081	0.250
SCIO	47.222	3.239	12.000	0.234
NDRC	47.222	36.129	12.000	0.178
CHINATAX	47.222	4.022	12.000	0.234
NMPA	47.222	3.239	12.000	0.234
MCT	44.444	7.533	11.960	0.216
CUSTOMS	44.444	3.079	11.960	0.223
NRTA	41.667	7.558	11.921	0.193
SPB	38.889	0.556	11.881	0.207
FORESTRY	36.111	0	11.842	0.197
CNIPA	36.111	0	11.842	0.197
NATCM	27.778	0	11.726	0.144
CBIRC	27.778	0	11.726	0.144
NHC	27.778	0	11.726	0.144
SAIC	27.778	0.571	11.726	0.129
AQSIQ	27.778	0.571	11.726	0.129
MOT	22.222	0	11.650	0.102
CFDA	22.222	0	11.650	0.102
SHDF	16.667	0	11.576	0.093
ADBC	8.333	0	11.465	0.031
WENMING	8.333	0	11.392	0.044
MOA	8.333	0	11.465	0.031
SASAC	8.333	0	11.392	0.029
MOF	8.333	0	11.392	0.029
CSRC	2.778	0	11.321	0.018
CAA	0	0	-	0
GOSCNPC	0	0	-	0
NPC	0	0	-	0
GOV	0	0	-	0
AMCSC	0	0	-	0
SAPPRFT	0	0	-	0
COURT	0	0	-	0

As shown in Fig 6 and Table 14, the structural characteristics of publishers such as CAC, MPS, PBC, and MIIT, are generally rated high. In terms of degree, CAC is rated the highest, followed by MPS, PBC, MIIT, MOFCOM, and SAMR, which are also rated above 0.5, indicating that these departments are the major forces in formulating and implementing relevant policies for the live streaming e-commerce industry. In terms of Betweenness, CAC is rated the highest, followed by the MIIT, the NDRC, the MPS, and the PBC, indicating that these departments can better coordinate with other departments in releasing and implementing policies, and communicate with various departments. In terms of closeness, CAC, MPS, PBC, MIIT, MOFCOM, and SAMR all have good performance, indicating that they cooperate with other departments quickly. In terms of eigenvector, CAC, MPS, PBC, MIIT, and SAMR are rated higher than other departments are, indicating that these departments are highly correlated with other core nodes and represent core nodes in the network. In summary, the entire social network model follows a multi-subject coordination pattern dominated by CACs, with MPS, PBC, MIIT, MOFCOM, SAMR and NDRC serving as local cores.

**Fig 7:**
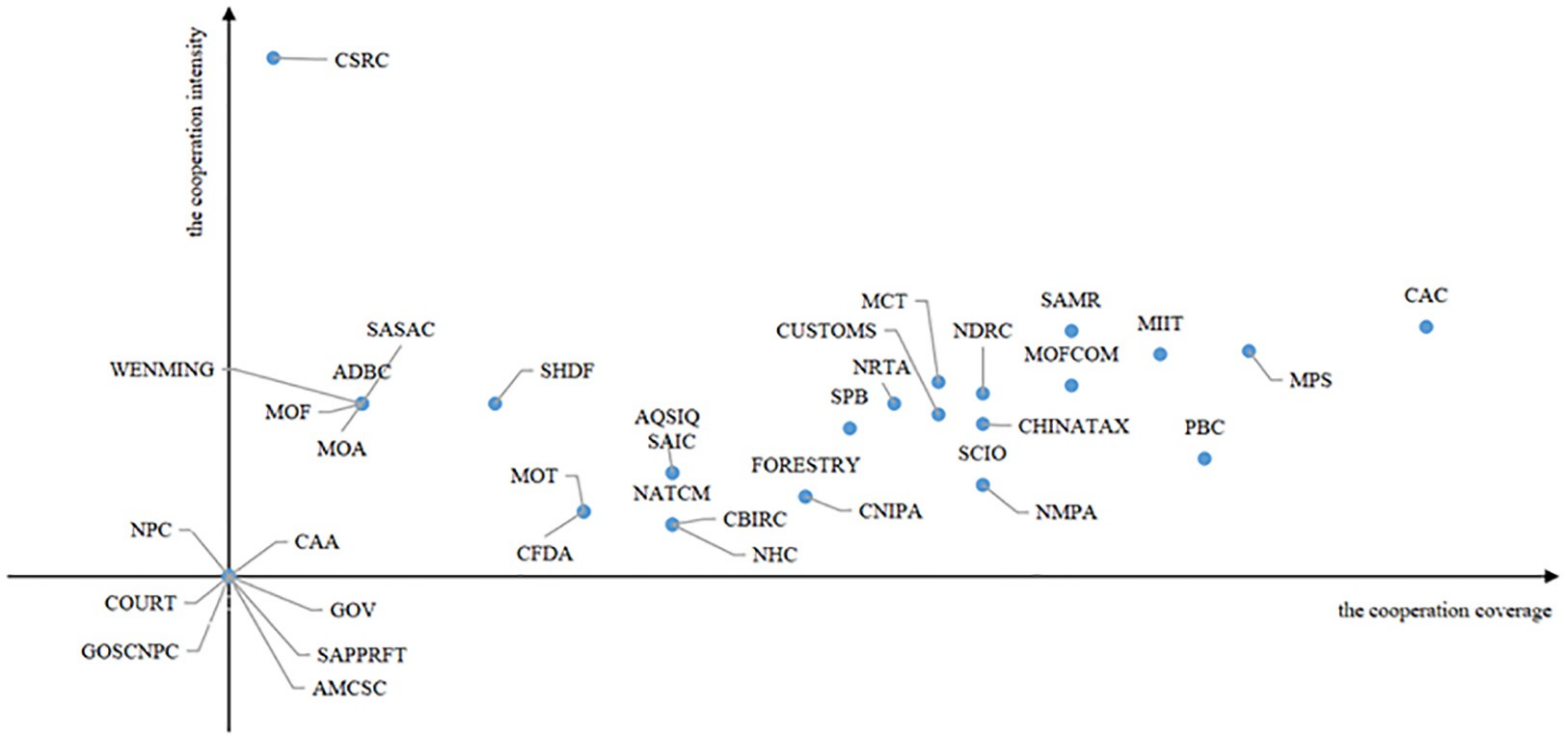
Cooperation matrix of policy publishers.

Finally, this paper further analyzes the network role characteristics of policy publishers from the perspectives of cooperation coverage and intensity. According to the research of Liu [27], cooperation coverage is defined as the number of departments cooperating with policy publishers, i.e., the number of nodes in the cooperation network. Furthermore, cooperation intensity is defined as the ratio of the number of policies jointly released by a policy publisher to the number of cooperative departments. If the ratio cannot be achieved, it is recorded as 0. Then, taking the cooperation coverage as the horizontal axis, the cooperation intensity as the vertical axis, and the median in the data set as the origin coordinate, the cooperation matrix of policy publishers is constructed. The results are shown in Fig 7.

As shown in Fig 7, there are 7 policy publishers with wide cooperation coverage and high intensity; these publishers belong to the wide coverage-high intensity entities, namely, CAC, SAMR, MPS, MIIT, MCT, MOFCOM and NDRC, indicating that they have carried out all-round, multi-level and wide-field in-depth cooperation with other policy publishers and are in a key position in the cooperation network. Moreover, CSRC, MOF, and WENMING have high cooperation intensities with other policy publishers, but the coverage is limited. SCIO, NMPA, FORESTRY, etc., cooperate with other entities. Their coverage is good, but the cooperation intensity is below average. Other policy publishers, such as NPC, GOV, and AMCSC, are poor in terms of cooperation intensity and coverage. This demonstrates that the current cooperation space among relevant policy publishers in the live streaming e-commerce industry urgently needs further development, and the relevant policy cooperation mechanism still needs to be improved.

In summary, the degree of synergy among the policy publishers in the live streaming e-commerce industry is strengthened, and the synergy density among the publishers is relatively high. However, synergistic departments are over-concentrated, and the policy functions and cooperation of other departments are too concentrated. The policy functions and cooperation ability of these countries have not been fully exerted. Therefore, government departments need to fully consider the complexity of the live streaming e-commerce industry and the diversity of public needs. As a new type of consumption model, the live streaming e-commerce industry plays a pivotal role in promoting the transformation and upgrading of the economy across all walks of life, accelerating the development of new business formats in the internet economy, and supporting the reform of cross-border e-commerce and poverty alleviation. Furthermore, due to the loose threshold, a large number of unemployed laborers joined during the epidemic, and countless unemployed people, trapped in the workplace, and blocked from entrepreneurship seized this opportunity to find a new way out of the predicament of failure. The live streaming e-commerce industry covers a wide range of fields, has many participants, and is deep, which also leads to complex industry structures and policy formulation difficulties. Accordingly, relevant policy formulation and implementation departments should enhance their co-governance capabilities, and establish the concept of policy coordination by building a cross-departmental policy formulation and release platform, thereby promoting communication and cooperation among departments, optimizing coordination mechanisms among departments, and improving the feasibility of policy implementation.

## 6. Conclusions

This paper collects a total of 59 policies related to the live streaming e-commerce industry released by various government departments at the national level from 2016 (when the live streaming e-commerce industry first appeared) to 2022. Quantitative analysis is carried out from the perspectives of policy tools, policy effect evaluation, and policy publishers to discuss the existing problems in current policies. This paper aims to provide guidance and suggestions for the Chinese government to further improve the rationality and effectiveness of the relevant policy system of the live streaming e-commerce industry, actively promote the healthy development of the live streaming e-commerce industry, and accelerate the adaptation of all walks of life to the new business format of the internet economy. The following conclusions are drawn from analysis.

1. From the perspective of policy tools, the overall structure of policy tools in the live streaming e-commerce industry is unreasonable, and different types of policy tools are significantly diverse. Among them, environment-based policy tools account for the highest proportion, reaching 62.97%, and instruments related to industrial norms and regulations account for the largest proportion of the country’s structure. This indicates that China has focuses mainly on various types of chaos in the industry. To protect the rights and interests of consumers, the industry’s normative and regulatory system should be continuously improved. However, it should be noted that the overflow of environmental policy tools and the weakening of supply-based and demand-based policy tools reflect that the government responds slowly to market demand. Enterprises, citizens, related organizations, and other diversified subjects fail to truly participate in the construction of the live streaming e-commerce industry, which weakens the government’s role in driving market demand and is not conducive to helping the role of live streaming e-commerce industry drive economic recovery during the epidemic. Moreover, to speed up economic transformation and upgrade and better adapt to the new business formats of the Internet economy, all walks of life join the new economic model of live streaming e-commerce, which will inevitably make their systems more tremendous and complicated. Therefore, in the formulation and implementation of government policies, the diversity of participants in live streaming e-commerce must be considered, and participants should communicate with relevant policy-making departments, associations, enterprises, and platforms in other industries while formulating policy plans. In addition, when focusing on environment-based policy tools and improving industry norms, the government should pay attention to the pulling force of supply-based and demand-based policies on the live streaming e-commerce industry, intensify the professional training of talent in the live streaming e-commerce industry, optimize the incentive system, ensure the intensity of capital investment, refine investment areas, strengthen the support of various production factors, and maintain enthusiasm for the live streaming e-commerce industry.
2. From the perspective of policy effectiveness evaluation, due to the frequent chaos in the live streaming e-commerce industry, government departments are paying more attention to the protection of consumer interests, resulting in industry normative and regulatory policies constituting the majority of policies. Therefore, the current effectiveness evaluation of relevant policy performance in the live streaming e-commerce industry is not excellent. Among the policies evaluated, only those related to the promotion of industrial development have higher scores. In addition, the scores of the first-level variables are also relatively balanced. This shows that there is still much room for improvement in policy design: on the one hand, most policies generally have single policy effectiveness, and the formulation of long-, medium- and short-term goals is not detailed, which reduces policy stability to a certain extent. On the other hand, the settings of policy priorities, incentives, and action modes are not perfect, indicating that the government has insufficient impetus and pulling power for the live streaming e-commerce industry. Therefore, while formulating relevant policies in the future, the government should formulate small goals in stages under the guidance of the overall goal; take the macro-level and micro-level of the policy into account; enrich the policy content; and consider financial subsidies, technical support, tax incentives, and talent incentives based on the national conditions and the specific situation within the industry.
3. From the perspective of policy publishers, the coordination of relevant policy publishers in the live streaming e-commerce industry is constantly intensifying. However, there is an over-concentration of coordination departments, mainly CAC, SAMR, MPS, MIIT, and other departments, while the policy functions and cooperation capabilities of other departments have not been fully exerted. In the future, an increasing number of industries and enterprises will join the live streaming e-commerce industry to promote their economic transformation and upgrading. Therefore, all government departments need to fully consider the complexity of the live streaming e-commerce structure and the diversity of public needs, establish the concept of cross-departmental policy cooperation, build a cross-departmental policy formulation and release platform, promote communication and cooperation among departments, and improve the coordination of policy implementation.

The paper has the following limitations, which need to be further explored:

1. An analysis of the current situation of the policies of the live streaming e-commerce industry reveals that most of the current industry policies are normative policies, but there is no in-depth discussion on whether they can effectively reduce the occurrence of industry chaos.
2. In this paper, the policy is merely analyzed from the perspective of textual quantification, and the social effects of policy implementation cannot be intuitively obtained. In a period when the live streaming e-commerce model is booming, the introduction of relevant policies will inevitably attract the attention of the general public and lead to heated discussions. The main topics that the public pays attention to before and after the implementation of the policy and the effect of policy implementation can be effectively analyzed through online public opinion. Therefore, in follow-up research, an in-depth analysis will be carried out on the social effects of implementing standardized policies in the live streaming e-commerce industry.
